# Trunk Impairment as a Predictor of Activities of Daily Living in Acute Stroke

**DOI:** 10.3389/fneur.2021.665592

**Published:** 2021-06-17

**Authors:** Masahiro Ishiwatari, Kaoru Honaga, Akira Tanuma, Tomokazu Takakura, Kozo Hatori, Akihiro Kurosu, Toshiyuki Fujiwara

**Affiliations:** ^1^Department of Rehabilitation Medicine, Juntendo University Graduate School of Medicine, Tokyo, Japan; ^2^Department of Rehabilitation, Kiminomori Rehabilitation Hospital, Chiba, Japan; ^3^Department of Physical Therapy, Juntendo University Faculty of Health Science, Tokyo, Japan

**Keywords:** acute stroke, trunk function, prediction, activities of daily living, trunk impairment scale

## Abstract

**Background and purpose:** Trunk function plays a key role in performing activities of daily living (ADL) including locomotion and sitting. Sitting and ADL should be performed as early as possible especially during the acute phase of stroke rehabilitation. Therefore, this study aimed to assess trunk function among patients with acute stroke using the Trunk Impairment Scale (TIS) and to predict its functional outcomes.

**Methods:** Overall, 67 patients with acute stroke (i.e., within 2 days of occurrence of the stroke) were included. The following clinical assessment items were obtained within 48 h after stroke onset and on the day before discharge from the hospital. Trunk function was examined using TIS and Trunk Control Test (TCT). The motor function of the upper and lower extremities was assessed using the stroke impairment assessment set motor (SIAS-M) score, and ADL was assessed using functional independence measure motor (FIM-M) items.

**Results:** Multiple regression analysis was performed using the stepwise regression method, using the total FIM-M score following discharge as the dependent variable and age, TIS, TCT, SIAS-M, and FIM-M within 48 h after stroke onset as the independent variables. Age, TIS, and FIM-M within 48 h after stroke onset were selected as the input variables and showed a high-adjusted determination coefficient (R^2^ = 0.79; *P* < 0.001).

**Conclusion:** TIS is a reliable method for evaluating trunk control function and is an early predictor of ADL among patients with acute stroke.

## Introduction

Trunk function is frequently impaired after stroke, affecting balance, gait, and activities of daily living (ADL) (1, 2). In stroke rehabilitation, trunk control is a fundamental motor skill that is essential for performing many functional tasks (3). In fact, the function of the trunk is not just ensuring the balance when sitting but also providing the ability to stabilize the proximal part of the body, allowing the movement of the distal part and selectively initiating trunk movements (4). There are several studies in the literature that investigated muscle strength in the extremities after stroke (5–7). There are also studies that have assessed the trunk muscle strength as the ability to control balance, trunk movement, and trunk muscle strength in the sitting and standing positions (1, 8–12). Verheyden et al. emphasize the importance of trunk performance, particularly that related to the static sitting balance, when predicting functional outcome after stroke (13). In the stroke rehabilitation process, the trunk function is an important predictor of the functional outcome (1, 8, 14). Therefore, the trunk function plays a key role in basic activities, such as sitting, transferring from the supine to the sitting position, and also rolling.

In acute stroke rehabilitation, it is important to prevent the decline of physical activity and to improve ADL. Acquisition of sitting ability and trunk performance are necessary to improve physical activity and ADL (2, 15). Fujiwara et al. developed the seven-item Trunk Impairment Scale (TIS) to assess trunk dysfunction in patients with stroke (3). Validity and reliability have already been examined. The TIS developed by Verheyden et al. was shown to be effective in predicting the functional outcome of subacute stroke (13). According to the Agency for Health Care Policy and Research guidelines, turning, sitting, and other activities should be started within 24–48 h after stroke onset, if medically possible (16). The group that started rehabilitation within 72 h of admission had a shorter length of stay and better walking status at discharge than the group that started rehabilitation >72 h after the admission (2). Although the mortality rate remained the same, the functional outcome tended to be better when patients increased the amount of training in the acute phase by starting sitting and standing rehabilitation within 24 h of the onset of illness (17).

van Nes et al. in their study of balance using individually adjustable chairs placed on a force platform considered 5–6 weeks after the onset as the subacute phase (18). Franchignoni et al. rather focused on patients with subacute stroke, with an average of 46 days between stroke onset and admission for rehabilitation (14). Other studies in the literature have included patients 1–2 weeks after stroke onset (19, 20) and patients who were transferred to a rehabilitation hospital 1–3 months after stroke onset, and these patients were able to maintain a sitting position (4, 9, 13, 18, 21). For the former reasons, we have classified the acute phase as within 2 weeks and the subacute phase as within 1–3 months after stroke onset. In our study, assessing trunk function with TIS within 48 h after stroke onset helped us assess the level of functional impairment in patients with stroke at the bedside in the acute phase, even if the patients were unable to safely maintain a seated position.

The most frequently identified variables predicting ADL after stroke include age and initial severity of motor and functional deficits (22). Trunk performance has also been identified as an important independent predictor of ADL after stroke (3, 9, 13, 22, 23). Fujiwara et al. conducted a multiple regression analysis to predict the Functional Independence Measure (FIM) motor score at discharge and confirmed that adding TIS as one of the predictors improved the explanation of variation in the FIM motor score at discharge from 66 to 75%, which can contribute to the prediction of functional status after stroke (3). Verheyden et al. examined the predictive validity of TIS and its subscales in predicting the Barthel Index score at 6 months after stroke onset in a multicenter study; the best predictors of the Barthel Index score were the TIS total score and the static sitting balance subscale score at admission (13). Collin and Wade (1) developed the Trunk Control Test (TCT) to assess the trunk function in patients with stroke. Franchignoni et al. (14) reported that using the TCT score at admission as one of the predictors better explains the FIM score at discharge than the FIM score at admission alone. The addition of trunk function assessment to ADL at discharge allowed the determination of a strong prognostic value. The clinical tools to assess the trunk performance include TCT (1, 14, 23), the trunk control items of the Postural Assessment Scale for Stroke (PASS) (9), TIS developed by Fujiwara et al. TIS (3), and TIS developed by Verheyden et al. (24). To better understand the recovery of the trunk function after stroke and to develop more effective treatment programs for patients with trunk imbalances, the trunk function needs to be assessed at the level of ability impairment and functional impairment.

A safe and less time-consuming evaluation method is desirable for patients with acute stroke. To the authors' knowledge, no previous study has reported the assessment of the trunk function and its prognosis within 48 h. Therefore, the purpose of this study was to investigate the prognosis prediction of patients with acute stroke using TIS for the assessment of the body trunk function.

## Subjects and Methods

### Subjects

The study subjects included 93 patients admitted for cerebral infarction or hemorrhage at an acute hospital in Chiba, Japan, with 115 beds, from March to September 2017. The inclusion criteria were as follows: first diagnosis of a unilateral stroke based on a confirmatory evidence from computed tomography or magnetic resonance imaging of the brain. The rehabilitation was started within 48 h after stroke onset, and the trunk function was assessed using TIS within 48 h after stroke onset. Medical stability was initiated under medical supervision and performed in accordance with the risk management for stroke. The level of consciousness was defined as being awake without stimuli but not being clearly conscious. The exclusion criteria were as follows: patients in whom clinical assessment was difficult due to disturbed consciousness, those who underwent surgery, those with stroke exacerbation, and those who died. A total of 67 (31 men and 36 women) were enrolled: 50 with cerebral infarction and 17 with cerebral hemorrhage. Their median age and length of stay (LOS) were 81 [interquartile range (IQR), 77–89] years and 21 (IQR, 16.5–26.5) days, respectively (Table 1). The discharge destination after acute hospital care was home for 12 patients, rehabilitation hospital for 48 patients, and nursing home for 7 patients. The participants received complete explanation of the study purpose and then provided written informed consent. For patients who were unable to sign their names, a family member or another authorized representative provided the written informed consent. The Ethics Review Committee of Shioda Memorial Hospital, Chiba, Japan, approved the study (Approval Number: 2). All study-related processes were performed in consonance with the principles of the Helsinki declaration.

**Table 1:** Participant Characteristics.

**Participant Characteristics**		
Number of participants (male/female)	67 (31/36)	
Age (years old, median, IQR)	81 (77–89)	
Length of stay (median days, IQR)	21 (16.5–26.5)	
**Diagnosis**		
Cerebral infarction	50 (75%)	
Cerebral hemorrhage	17 (25%)	
Paretic side right/left	33/34	
	Admission	Discharge
TCT (median IQR)	36 (12–61)	74 (37–100)
TIS (median IQR)	7 (3–12)	14 (10–17)
SIAS-M (median IQR)	10 (4–15)	16 (7–18)
FIM-M (median IQR)	18 (13–26)	38 (23–61)

*TCT, Trunk Control test; TIS, Trunk Impairment scale; SIAS-M, Stroke Impairment Assessment Set motor score; FIM-M, Functional Independence Measure motor score; IQR, interquartile range*.

### Methods

Basic information, such as diagnosis, disability side, age, sex, and LOS, was collected. TIS and TCT were used to assess the trunk function, the SIAS (10) motor items (SIAS-M) were used to assess the motor function on the paralyzed side, and the Functional Independence Measure motor (FIM-M) items (25) were used to assess ADLs. The level of consciousness was defined as being awake on stimulation and easily opening the eyes on normal calling. The same examiner performed the assessment within 48 h after stroke onset as the initial assessment and the day before discharge as the final assessment day.

### Trunk Function Assessment

#### TIS

The perception of trunk verticality and the recovery response to stabilize the body play important roles in maintaining the sitting position (26, 27). Furthermore, turning over and the transfer from the supine to the sitting position require the activation of the abdominal muscles, which are the main movers of trunk flexion, in a chain that includes the postural synergy of the iliopsoas and rectus femoris muscles (27–29). These are the reasons why Fujiwara et al. developed TIS, which consisted of seven items with a maximum score of 21 points, with higher scores indicating higher trunk ability. The items related to the strength of the abdominal muscle and those related to verticality were obtained from SIAS. The other five items developed for TIS, namely, perceived trunk verticality, rotational trunk strength on the side affected as well as the unaffected side (ROT-A and ROT-U), and righting reflex on the affected and unaffected sides (RR-A and RR-U), have been shown to have high reliability, validity, and responsiveness (Table 2) (3).

**Table 2:** Trunk Impairment Scale Items and Scoring Criteria.

**Item**	**Method**	**Scoring**
Perception of trunk verticality	•While the patient is sitting on the edge of a bed or on a chair without a backrest, with the feet off the ground, the examiner holds both sides of the patient's shoulders and makes the patient's trunk deviate to the right and left. •The examiner asks the patient to indicate when he or she feels the trunk is in a vertical position. •The examiner then records the degree of trunk angle deviation from the vertical line drawn from the midpoint of the Jacoby line.	•0: The angle is ≥30°. •1: The angle is <30° and ≥20°. •2: The angle is <20° and ≥10°. •3: The angle is <10°.
Trunk rotation muscle strength on the affected side	•The patient is asked to roll the body from the supine position to the unaffected side. •The arms should be crossed in front of the chest and legs kept extended. •The patient is asked to roll his or her body without pushing the floor with his or her limbs or pulling on bed clothes. •Isometric contractions for stabilization and other muscles than external oblique (e.g., pectoralis major) activation during rolling are allowed.	•0: No contraction is noted in external oblique muscles on the affected side. •1: External oblique muscle contraction is visible on the affected side, but the patient cannot roll his or her body. •2: The patient can lift the affected side scapula but cannot fully rotate the body. •3: The patient can fully rotate the body.
Trunk rotation muscle strength on the unaffected side	•The patient is asked to roll the body from the supine position to the affected side.	•Scoring is the same as for the trunk rotation muscle strength on the affected side.
Righting reflex on the affected side	•The patient sits on the edge of a bed or a chair without a backrest. •The examiner pushes the patient's shoulder laterally (about 30 degrees) to the unaffected side and scores according to the degree of the reflex elicited on the affected side of the patient's trunk.	•0: No reflex is elicited •1: The reflex is poorly elicited, and the patient cannot bring his or her body back to the erect position as before. •2: The reflex is not strong, but the patient can bring his or her body back to the erect position almost as before. •3: The reflex is strong enough, and the patient can immediately bring his or her body back to the erect position as before.
Righting reflex on the unaffected side	•The examiner pushes the patient's shoulder laterally (about 30 degrees) to the affected side.	•Scoring is the same as for the righting reflex on the affected side.
Stroke impairment assessment set verticality	•Instruct the patient to remain in the sitting position.	•0: The patient cannot maintain the sitting position. •1: A sitting position can only be maintained while tilting to one side, and the patient is unable to correct the posture to an erect position. •2: The patient can sit vertically when reminded to do so. •3: The patient can sit vertically in a normal manner.
Stroke impairment assessment set abdominal muscle strength	•Stroke Impairment Assessment Set abdominal muscle strength is evaluated with the patient resting in a 45° semireclining position in either a wheelchair or a high-back chair. •The patient is asked to raise the shoulders off the back of the chair and assume the sitting position.	•0: Unable to sit up. •1: The patient can sit up provided there is no resistance to the movement. •2: The patient can come to the sitting position despite pressure on the sternum by the examiner. •3: The patient has good strength in the abdominal muscles and is able to sit up against considerable resistance.

*Source: Am J Phys Med Rehabil (Fujiwara T, et al., 2004, 83:681-8)*.

*Created by citing the development of a new scale for assessing trunk impairment after stroke (Trunk Impairment Scale). Its psychometric properties (2004) (3)*.

*Reprinted with permission of the original author Dr. Toshiyuki Fujiwara, Senior Professor*.

#### TCT

Overall, the four simple aspects involved in the trunk movement were evaluated using TCT. This method examines the maintenance of the sitting position, the ability to roll from the supine position toward the affected and unaffected sides, and the transfer from the supine to the sitting position. The scoring for this exercise has three assessment levels: 0, 12, and 25 points for each level. The perfect score is 100, and the higher the score, the better the trunk ability. High reliability and validity have been confirmed by Collin and Wade (1).

### Paraplegic Motor Function Assessment

SIAS-M was used for paralytic side motor function. SIAS consists of 22 items: 5 on proximal and distal motor function, 4 on tendon reflexes and muscle tone, 4 on superficial and deep sensation, and 9 on other range of motion, pain, trunk, higher brain, and unaffected side function (10). SIAS-M evaluates the proximal and distal motor function of the upper and lower extremities on a 25-point scale of five items, with a high score indicating high motor function.

### ADL Assessment

ADL is assessed using exercise items of FIM: (1) eating; (2) dressing; (3) wiping; (4) change of clothes for upper body; (5) change of clothes for lower body; (6) using the toilet; (7) urinating; (8) defecating; (9) transferring to a bed, chair, or wheelchair; (10) transferring to the toilet; (11) transferring to the bathtub; (12) walking; and (13) climbing stairs. It is rated on a seven-point scale from maximum assistance (one point) to complete independence (seven points), with a higher score on a 91-point scale indicating more independence in the daily living (25).

### Risk Management

The general principles are as follows: (a) wakefulness without stimulation, but without clear consciousness; (b) the neurological condition must not have worsened within 24 h of admission; and (c) there must be no contraindication to cardiac exercise.

The criteria for starting weaning according to the type of disease and condition are as follows: (1) cardiogenic cerebral infarction: weaning after confirming the presence of atrial thrombus and cardiac function by transthoracic echocardiography; (2) atherothrombotic cerebral infarction: weaning after confirming the general condition and the presence of stenosis and plaque in the main artery by magnetic resonance angiography or carotid echocardiography; (3) lacunar infarction: weaning from the day of diagnosis; and (4) cerebral hemorrhage: release if there is no increase in hematoma and the development of hydrocephalus on follow-up head computed tomography.

The criteria for discontinuation are as follows: (1) cerebral infarction: systolic blood pressure of ≥200 mmHg and a fall of ≥20-30 mmHg from rest; (2) hemorrhagic stroke, ≥3-180 mmHg; (3) intracerebral hemorrhage, ≥5-200 mmHg; (4) a heart rate max with ≥120 beats per minute; and (5) oxygen saturation: an oxygen saturation of <92%.

### Statistics Analysis

For statistical analysis, the Shapiro–Wilk test was performed prior to each test to determine if each variable followed a normal distribution. Diagnosis, disability side, and sex were converted to dummy variables for nominal scales: diagnosis (cerebral infarction, 0; cerebral hemorrhage, 1), disability side (left, 0; right, 1), and sex (female, 0; male, 1). To determine multicollinearity, the FIM-M at discharge and Spearman's rank correlation coefficients for each item were analyzed, and variables with absolute values of correlation coefficients |r| of ≥0.9 were examined. A stepwise multiple regression analysis was performed with FIM-M at discharge as the dependent variable and age with significant correlation, TIS, TCT, SIAS-M, and FIM-M within 48 h after stroke onset (admission) as the explanatory variables. In the explanatory variables of the obtained multiple regression equations, whether no variables had a variance inflation factor of ≥10 was determined. Next, to predict the FIM-M at discharge, predictions and the correlation coefficient with FIM-M at discharge were calculated. Finally, the Kruskal–Wallis test was used to study if there was a significant difference between the ability to hold a seated position at discharge and TIS at discharge. SPSS version 25 was used for the statistical processing. The significance level was set at <5%.

## Results

### Concurrent Validity

The Spearman's rank correlation coefficient between the TIS and TCT scores was 0.90 (*P* < 0.001), indicating high concurrent validity. The Spearman's rank correlation coefficients between FIM-M and each item at discharge are shown in Table 3.

**Table 3:** Spearman's rank correlation coefficient (FIM-M at discharge and each item).

	**Discharge FIM-M**	**Age**	**Admission FIM-M**	**TIS**	**TCT**
Discharge FIM-M					
Age	−0.47^**^				
Admission FIM-M	0.87^**^	−0.36^**^			
TIS	0.82^**^	−0.28^*^	0.82^**^		
TCT	0.81^**^	−0.28^*^	0.86^**^	0.90^**^	
SIAS-M	0.81^**^	−0.30^*^	0.85^**^	0.90^**^	0.89^**^

*FIM, Functional Independence Measure; TIS, Trunk Impairment Scale; TCT, Trunk Control Test; SIAS-M, Stroke Impairment Assessment Set-Motor*.

** *P < 0.01*.

* *P < 0.05*.

### Predictive Validity

Multiple regression analysis was performed using the stepwise variable method. The total value of the FIM-M at discharge served as the dependent variable, with age, TIS, TCT, SIAS-M, and FIM-M within 48 h after stroke onset (admission) as the independent variables. Thus, TIS and FIM-M at admission were adopted, but TCT and SIAS-M at admission were excluded. A high R^2^ value (0.79; *P* < 0.001) was obtained as the adjusted determination coefficient. To predict FIM-M at discharge, a prediction formula was derived based on the findings of the multiple regression analysis (Table 4). The predicted FIM-M value at discharge was calculated using the following prediction formula:

**Table 4:** Result of Stepwise Multiple Regression Analysis.

		**Nonstandard coefficient**		**Standard Coefficient**			
**Dependent variable**	**Independent variables analyzed**	**B**	**SE**	**β**	***t*-value**	***P*-value**	**Adjusted R2**
Discharge FIM-M score	Constant	46.954	9.304		5.046	<0.001	
	Age	−0.43	0.104	−0.246	−4.146	<0.001	
	Trunk Impairment Scale (TIS) admission	1.874	0.365	0.488	5.127	<0.001	0.79
	FIM-M admission	0.699	0.193	0.342	3.617	0.001	

*FIM-M, Functional Independence Measure Motor score*.

*Predicted value of FIM-M at discharge = constant 46.954 + (1.874 × TIS admission) + (−0.430 × age) + (0.699 × FIM-M admission)*.

Predicted FIM-M value at discharge = constant 46.954 + (1.874 × TIS admission) + (−0.430 × age) + (0.699 × FIM-M admission).

**Figure 1:**
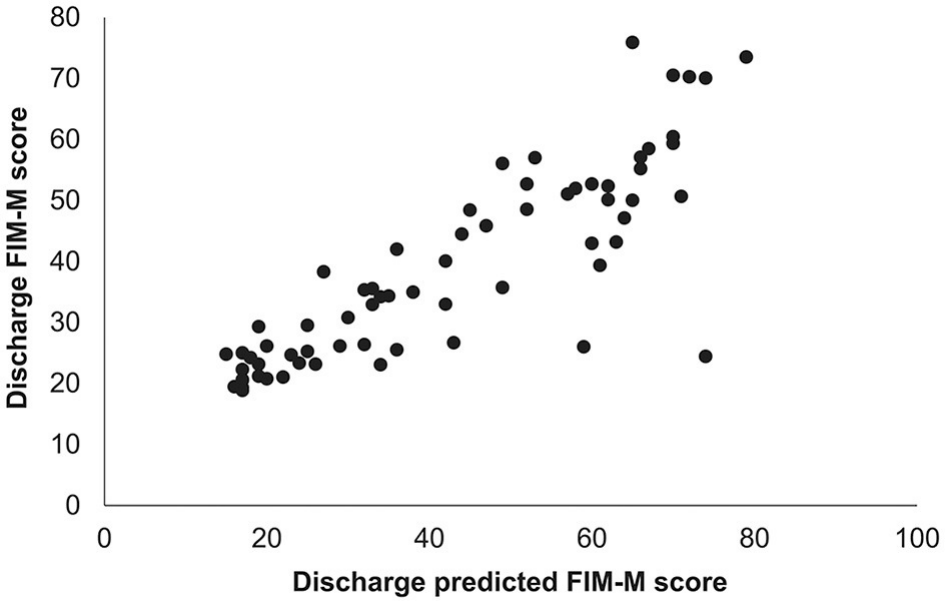
Relationship between predicted and measured FIM-M scores at hospital discharge. The predicted FIM-M value at the time of hospital discharge was calculated using a prediction equation based on the results of multiple regression analysis. A high correlation was found between the predicted FIM-M value and the actual FIM-M value at the time of hospital discharge (Spearman's rank correlation coefficient = 0.89, *P* < 0.001). FIM-M, Functional Independence Measure motor score.

A high correlation was observed between the obtained predicted FIM-M and the actual FIM-M value at discharge (r = 0.89; *P* < 0.001; Figure 1).

### Sitting Position at Discharge

At discharge, 14 (20.9%) patients had difficulties in maintaining the sitting position, 25 (37.3%) patients could sit upright if instructed, and 28 (41.8%) patients could maintain the sitting position. The Kruskal–Wallis test showed a significant difference between the sitting position at discharge and TIS at discharge (*P* < 0.001).

## Discussion

The aim of this study was to predict the prognosis of patients with acute stroke after hospital discharge based on the TIS score. The results suggest that age, TIS within 48 h after stroke onset, and FIM-M can be used to predict FIM-M values at the time of hospital discharge.

Recently, the importance of early rehabilitation has been recognized, and very early intervention is said to have a significant impact on prognosis (30–34). The recovery of static sitting balance control in the subacute phase of stroke was studied using the force platform method, which accurately assesses changes in ground reaction forces during various sitting tasks in patients with stroke (22). In the unstable state, there was a clear improvement in the sitting balance in both directions of rocking, with the greatest improvement on average in the lateral direction. The significant effect of visual deprivation on lateral balance simply showed a decreasing trend, probably related to sensory reorganization, and the same pattern of recovery was observed for standing balance after acute stroke (35, 36). The impairment of the lateral sitting and standing balance is a characteristic sequela of the stroke. The loss of the hip abductor and adductor muscles is an important factor in the standing balance after stroke (37), but it has been suggested that the most important factor explaining the problems with the sitting balance is loss of the trunk. These studies were the first evidence for the idea that the selective muscle control of the trunk, which is initially impaired, can be restored after stroke (38, 39). The role of the compensatory activation of uncrossed pathways has been suggested for the recovery of the trunk function after stroke (40). In terms of the paralyzed side and trunk function, the lateral motor control system (i.e., lateral corticospinal tract and red nucleus tract) controls the distal limb movements and the motor movements of the fingers and sensory input. The medial motor control system (i.e., corticospinal tract, lateral vestibulospinal tract, and anterior corticospinal tract) controls the trunk and proximal muscles involved in standing, walking, postural reflexes, parallel function, and muscle tone (41). Even if only the external motor control system function is impaired, the ADL function cannot be achieved if the internal motor control system does not function because it is the basis of postural control. Although many reports have already demonstrated the motor function of the limbs (5–7), we believe that the severity and improvement of paralysis do not necessarily correspond to trunk function improvement. Several systematic reviews have investigated a method of measuring the balance in the clinical practice and as a clinical measurement tool for assessing the trunk function after stroke (42–44). TCT is the first clinical tool to assess the motor capacity of the trunk (1). The static and the dynamic balance, walking speed, walking distance circumference, LOS, and functional motor status at discharge during the inpatient rehabilitation of patients with hemiplegia and stroke were examined for determining the predictive value of TCT on admission. TCT correlates with FIM on admission and discharge the predictive power of TCT, as a single test, accounting for 52% of the variation in the LOS in rehabilitation and 54% of the FIM on discharge (23). However, TCT does not necessarily assess specifically the recovery of trunk dysfunction but rather the improvement of trunk function at the level of disability, as the movements are often performed using the non-impaired side to compensate for the loss of the trunk function. PASS examines the ability to maintain or change the posture in the supine, sitting, and standing positions (45). A study examining the relationship between the trunk control at 14 days after stroke onset and the overall ADL function in patients at 6 months after stroke onset, which used the PASS–trunk control (PASS-TC) items, found that the PASS-TC score to predict overall ADLs and its psychometric properties were well-confirmed by its ease of use in the clinical practice and research settings. The ability of the sit-and-reach test (SRT) to predict mobility in patients in the early acute phase of stroke was investigated. The distance reached on the SRT correlated with the FIM mobility score at discharge (r = 0.572, *P* < 0.002) and with the distance achieved on the timed walking test (r = 0.524, *P* < 0.006). To use the sitting balance as a predictor of outcome, it is essential to accurately measure the sitting balance in the acute phase of stroke. The SRT can be used as a quick screening tool with simple instructions to follow, requiring 1 min of sedentary holding for the implementation (20). For patients with acute stroke, the Function in Sitting Test, a simple test of sitting balance, has high internal consistency and was confirmed for content and construct validity by the item response theory analysis. The concordance was supported by the high correlations with the Modified Rankin Scale, static balance index, and the dynamic balance grade (21). The original version of the TIS was developed by Verheyden et al. in 2004. The test–retest and the inter-rater measurement error, internal consistency, construct, and concurrent validity were shown to be excellent (24). The total TIS score on admission and the score on its “static sitting balance” subscale were shown to be the best predictors of the Barthel Index score at 6 months after stroke (13). The TIS developed by Fujiwara et al. consists of seven items: two items derived from the Stroke Impairment Assessment Set (10) (“Abdominal Strength” and “Verticality”) and five items originally developed for the TIS (“Perception of Trunk Verticality,” “Trunk Rotation Strength” on the affected and unaffected sides, and “Righting Reflex” on the affected and unaffected sides). While both TISs conducted extensive testing of essential psychometric properties, this is lacking in TCT. The TIS of Verheyden et al. has been evaluated for ceiling effects, but no Rasch analysis has yet been performed (4). The TIS of Fujiwara et al. has been evaluated by the Rasch analysis, but the ceiling effect has not yet been assessed (3). Each assessment of the trunk function has been tested for reliability and validity. However, they also include the assessments of static sitting position and balance. If the patient is unable to maintain the sitting position, it is difficult to continue the assessment. While PASS-TC and SRT have been tested in patients with acute stroke, the present study was conducted as early as 48 h after stroke onset using TIS. To better understand the recovery of the trunk muscle function after stroke and to develop an effective treatment strategy for the people who develop disabilities associated with the trunk imbalance, it is necessary to assess the trunk function at both disability and ability levels. Although the early weaning intervention is performed in the acute phase in consideration of the risk management, the objective of this evaluation of the trunk function using TIS within 48 h after stroke onset is useful not only for the prognosis but also for the identification of the patient's potential and to plan a training program in a rehabilitation hospital. TIS seeks to assess the trunk function at a functional level, including trunk verticality recognition, recovery response, and trunk rotation strength. The bases for the use of these TIS items are the perception that trunk verticality is believed to be very crucial to maintain the vertical position, the capacity to evoke righting reflexes is considered very important for sustaining dynamic sitting balance, and the strength of the abdominal muscle is considered indispensable in sitting up from the lying position and in rotating the body. These elements consider the functional aspects. Although there is no gold standard for assessing the trunk function, TCT is one of the effective tools for assessing the trunk function after stroke. TIS (24), developed by Verheyden et al., is also widely used to assess the trunk function, as is TCT. The TIS consists of three subscales, static sitting balance, dynamic sitting balance, and coordination, which have been verified for reliability, internal consistency, and validity. However, a score of zero on the first item results in a total score of zero on TIS. In this study, we included patients within 48 h after stroke onset who were awake without stimulation but had no clear consciousness. TIS developed by Fujiwara et al. can be used to evaluate patients who have difficulty maintaining a sitting position. We believe that it is difficult to perform a functional assessment with the TIS of Verheyden et al. for patients who have difficulty maintaining a sitting position. Therefore, no comparison or examination was conducted in this study.

In a previous study by Fujiwara et al., TIS correlated well with TCT (Spearman rank correlation coefficient, 0.91), supporting its validity (3). In the present study, TIS used in the acute phase also correlated well with TCT (Spearman rank correlation coefficient, 0.90), supporting the validity of the agreement between the two. At the time of discharge, 14 (20.9%) patients had difficulty in maintaining the sitting position, 25 (37.3%) patients were able to sit vertically on instruction, and 28 (41.8%) patients were able to maintain the sitting position. When we examined whether there was a difference among the three groups, we found a significant difference (*P* < 0.001). These results suggest that TIS is a valid tool for assessing the trunk function. Using stepwise multiple regression analysis selected age, FIM-M, and TIS as explanatory variables, showing an adjusted R^2^ of 0.79. Our results showed that the TIS is a good predictor of ADL even in the acute phase of stroke rehabilitation. In this study, the correlation between predicted and measured FIM-M values at the time of discharge was very high (r = 0.89), which was favorable based on the multiple regression equation obtained with multiple regression analysis using the stepwise method. Using the multiple regression equation obtained in this study to understand early ADL abilities, training programs may be possibly designed to focus on the trunk function from the bed for patients with sitting difficulty.

TIS can be safely used at the bedside in patients with acute phase of stroke and can be used in clinical practice to assess the trunk function immediately after the onset of stroke. Results showed that the trunk function affects the ADL prognosis and that TIS is useful for predicting ADL prognosis in the acute phase of stroke.

### Limitations

The prognostic FIM-M value at discharge from an acute hospital was examined. However, the long-term outcome was not assessed. Therefore, the predictive validity of TIS should be assessed in the acute phase for long-term FIM-M, such as that at 1 year after stroke onset. Cognitive function is one of the important predicting factors for ADL. However, cognitive function details were not determined. Therefore, further study should be examined to examine the cognitive function and long-term functional outcome.

## Data Availability Statement

The raw data supporting the conclusions of this article will be made available by the authors, without undue reservation.

## Ethics Statement

The studies involving human participants were reviewed and approved by the ethical review board of our hospital Shioda Memorial Hospital Ethics Review Committee approved the study (Approval Number: 2). The patients/participants provided their written informed consent to participate in this study.

## Author Contributions

MI: conceptualization, formal analysis, methodology, data curation, investigation, resources, writing—original draft preparation, and writing—reviewing and editing. KHo: conceptualization, formal analysis, investigation, methodology, software, and validation. AT: conceptualization, formal analysis, methodology, software, and validation. TT: conceptualization, investigation, methodology, and validation. KHa: conceptualization, methodology, and validation. AK: conceptualization, methodology, software, and validation. TF: conceptualization, formal analysis, methodology, software, supervision, visualization, project administration, writing—original draft preparation, and writing—reviewing and editing. All authors contributed to the article and approved the submitted version.

## Conflict of Interest

The authors declare that the research was conducted in the absence of any commercial or financial relationships that could be construed as a potential conflict of interest.
